# A Simple Model for the Evaporation of Hydrometeors and Their Isotopes

**DOI:** 10.1029/2024JD041126

**Published:** 2024-11-20

**Authors:** Simon P. de Szoeke, Mampi Sarkar, Estefanía Quiñones Meléndez, Peter N. Blossey, David Noone

**Affiliations:** ^1^ College of Earth, Ocean, and Atmospheric Sciences Oregon State University Corvallis OR USA; ^2^ Institute of Climate and Atmospheric Science University of Houston Houston TX USA; ^3^ Department of Earth and Atmospheric Sciences University of Houston Houston TX USA; ^4^ University of Washington Seattle WA USA; ^5^ Department of Physics University of Auckland Auckland New Zealand

**Keywords:** rain drop evaporation, stable water isotopes, isotope sources, numerical model, kinetic fractionation, rain drop size distribution

## Abstract

Cloud condensation and hydrometeor evaporation fractionate stable isotopes of water, enriching liquid with heavy isotopes; whereupon updrafts, downdrafts, and rain vertically redistribute water and its isotopes in the lower troposphere. These vertical water fluxes through the marine boundary layer affect low cloud climate feedback and, combined with isotope fractionation, are hypothesized to explain the depletion of tropical precipitation at higher precipitation rates known as the “amount effect.” Here, an efficient and numerically stable quasi‐analytical model simulates the evaporation of raindrops and enrichment of their isotope composition. It is applied to a drop size distribution and subcloud environment representative of Atlantic trade cumulus clouds. Idealized physics experiments artificially zero out selected processes to discern the separate effects on the isotope ratio of raindrops, of exchange with the environment, evaporation, and kinetic molecular diffusion. A parameterization of size‐dependent molecular and eddy diffusion is formulated that enriches raindrops much more strongly (+5‰ for deuterated water [HDO] and +3.5‰ for $H_{2}^{18}$O) than equilibrium evaporation as they become smaller than 1 mm. The effect on evaporated vapor is also assessed. Rain evaporation enriches subcloud vapor by +12‰ per mm rain (for HDO), explaining observations of enriched vapor in cold pools sourced by evaporatively cooled downdrafts. Drops smaller than 0.5 mm evaporate completely before falling 700 m in typical subtropical marine boundary layer conditions. The early and complete evaporation of these smaller drops in the rain size distribution enriches the vapor produced by rain evaporation.

## Key Points

- Evaporated vapor from trade cumulus rain is near the rain's initial isotope ratio and strongly enriched compared to surrounding vapor
- Rain isotope composition equilibrates to surrounding environmental vapor as it falls 10s of meters
- Before the smallest raindrops evaporate completely, molecular diffusion enriches them 40% more strongly than millimeter‐sized drops

## 1 Introduction

Shallow marine cumulus clouds cover much of the world's oceans and play a crucial role in the Earth's climate. In some climate models, marine cumulus cloud layers become desiccated by vertical mixing. These models have less clouds in a warmer climate, strong positive shallow cumulus cloud feedback, and increased climate sensitivity (Brient & Bony, 2013; Sherwood et al., 2014; Vial et al., 2017). The goal of Elucidating the Role of Clouds‐Circulation Coupling in Climate—Atlantic Tradewind Ocean Mesoscale Interaction Campaign (EUREC4A‐ATOMIC, Stevens et al., 2021) is to improve representation of water vapor fluxes in the trade cumulus layers of climate models. Observations of cloud amount increasing with cloud base mass flux in EUREC4A‐ATOMIC argue that the desiccation effect of vertical mixing is weaker than moistening by mesoscale circulations within the marine boundary layer that supply sub‐cloud layer vapor to the cloud layer (Vogel et al., 2022). Embedded within these cloud effects, the vertical profile of moisture is affected by the details of cloud detrainment and precipitation, including the focus of this study, hydrometeor reevaporation.

Cloud and precipitation processes affect the stable isotopologues of water—$H_{2}^{18}$O containing the heavy oxygen‐18 (^18^O) isotope, and deuterated water HDO containing the heavy deuterium (D or ^2^H) isotope. When phase changes between liquid and vapor occur, the heavy and light isotopes fractionate unequally among the phases. Water vapor is depleted of the rare heavy isotopes by fractionation in condensation near thermodynamic equilibrium and then removal of hydrometeors by precipitation, resulting in depletion with height of both tropospheric water vapor and depletion of the cloud liquid formed from it. Falling rain and downdrafts transport this depleted water downward. Converse to cloud condensation depleting rising vapor, hydrometeor evaporation enriches falling liquid because of the combined effects of equilibrium fractionation and differing molecular diffusion of light and heavy isotopes (i.e., kinetic fractionation). Intuition built on Rayleigh distillation is inaccurate: the sign of the effect of reevaporation on rain isotopic composition depends on the depletion of the ambient vapor relative to the rain, as well as the fraction of the drops evaporated.

Although the relationship between condensation and the isotopic composition of precipitation and vapor motivates their use as climate proxies, the problem of inferring the history of water from observed or modeled isotopes (Crawford et al., 2017; Dansgaard, 1964; Gat, 1996; Noone, 2012; Tremoy et al., 2014) is confounded by ambiguous combinations of multiple sources and processes (Galewsky et al., 2016; Hiron & Flossmann, 2020). One climate relationship involving water isotopes in the tropics is the observation that the precipitation ^18^O ratio is depleted of heavy isotopologues in months of stronger rain. Dansgaard (1964) called this correlation the “amount effect” (see also Rozanski et al. (1993)). Precipitation and near‐surface vapor can be depleted by horizontal import of depleted vapor when column precipitation exceeds evaporation (Lee et al., 2007; Moore et al., 2014), by organized mesoscale convective systems compared to isolated convection (Kurita, 2013), and by evaporation and exchange between rain and water vapor in unsaturated downdrafts (Bony et al., 2008; Risi et al., 2008). In high‐resolution simulations, the subcloud *vapor* is depleted by preferential export of enriched vapor by updrafts (Risi et al., 2020). Since rain reevaporation affects the isotopes of precipitation and vapor in tropical convection and may contribute to the amount effect (e.g., Risi et al., 2008), models of rain evaporation can be used to help interpret the isotope ratio of precipitation in past and present climates.

Isotope‐enabled general circulation models (Hoffmann et al., 1998; Noone & Simmonds, 2002; Sengupta et al., 2023; Steen‐Larsen et al., 2017; Werner et al., 2011) are used to estimate the effect of changes in the hydrological cycle on stable isotopes of water, but they parameterize cloud and precipitation processes, including vertical transport and mixing by convection and turbulence. Few isotope‐enabled general circulation models reproduce the observed relationship (Aggarwal et al., 2016) of isotopes to stratiform precipitation (J. Hu et al., 2018).

The finer grids in isotope‐enabled cloud resolving and large eddy simulation (LES) models (Blossey et al., 2010; Z. Hu et al., 2024; Risi et al., 2020, 2021; Wei et al., 2018) can explicitly represent the larger scales of convective and turbulent motions associated with clouds and convection, but parameterize cloud and rain microphysics. Torri (2022), for example, used such an approach to study rain isotopic composition in simulations of the isotopic composition in tropical deep convective precipitation, finding that the effect of rain evaporation is stronger than drop‐vapor exchange toward thermodynamic equilibrium. However, these LES often employ bulk microphysical schemes and assume a uniform isotopic content for all hydrometeors within a particular category, such as cloud droplets or raindrops (e.g., Blossey et al., 2010), when actually the rain isotopic composition varies with droplet size (Hiron & Flossmann, 2020).

Hydrometeors evaporate on time scales much shorter than resolved by the fluid dynamical time steps of LES (∼1 s). Standalone microphysical models (Graf, 2017; Graf et al., 2019; Salamalikis et al., 2016; Sarkar et al., 2023) integrate the time‐dependent equations for drop mass and isotopic composition across a distribution of falling and evaporating raindrops in a prescribed environment of surrounding vapor. These models allow the isotopic composition to vary across drop sizes in response to size‐dependent rain‐vapor exchanges. Although neglecting the joint evolution of rain evaporation and buoyancy‐driven turbulent fluid motion, such microphysical models complement high‐resolution models with simpler bulk microphysics by showing how size‐dependent processes affect the isotopic composition of vapor and rain.

This paper presents a simple algebraic model for rain evaporation and its isotopic content that illustrates the effects of turbulent and molecular diffusion, exchange with the surrounding vapor, and evaporation. We revisit algebraic solutions (Abraham, 1962; Best, 1952; Stewart, 1975) of the Craig and Gordon (1965) equation for the isotope ratio adapted for evaporating spheres. Drop ventilation factors that predict kinetic fractionation on microphysical time scales are based on lab experiments on falling drops (Gunn & Kinzer, 1949; Kinzer & Gunn, 1951; Stewart, 1975). We use these lab results to formulate a kinetic fractionation coefficient that can be used in the Craig and Gordon (1965) and Stewart (1975) solution for isotope ratio. This algebraic solution efficiently models the evaporation on time scales of less than 1 s of drops smaller than $10^{-6}$ m. Under the effect of the changing surroundings as drops fall through their environment, we simulate the mass and isotope ratios for a drop size distribution (DSD), and the isotope ratio of the vapor lost by the drops. We further use the model for idealized experiments to isolate the effects of kinetic fractionation and exchange with surrounding vapor, respectively.

Observations of stable isotopes of water in rain and vapor in cold pools (Bailey et al., 2023; Quiñones Meléndez et al., 2022; Sarkar et al., 2023) define cases and verification for our simulations in Section 2. The model for evaporation of atmospheric hydrometeors that solves for isotope concentration of raindrops and of evaporated vapor from the drops is presented in Section 3. Section 4 presents results of the model. The solutions show two regimes: In the *falling* regime, a small fraction of the drop evaporates as it falls through the environment. In the *vanishing* regime, the remaining hydrometeor completely evaporates as its fall speed goes to zero. A simulation is used to interpret rain and downdraft vapor observed during the EUREC4A‐ATOMIC field experiment. Sensitivity experiments demonstrate the effects of the DSD and environment on the isotopes of the evaporated vapor. In addition, in Section 4, illustrative physical experiments show the distinct contributions of exchange with surroundings, equilibrium fractionation, and differential diffusion. Section 5 summarizes the article.

## 2 Observations for a Cold Pool Case

Among the processes contributing to the isotope ratio of precipitation and water vapor, a subset of processes contributes to warm (nonfreezing) marine trade cumulus clouds, allowing us to isolate the effect of local water vapor sources. We simulate the hydrometeor evaporation leading to an atmospheric cold pool observed under tropical winter shallow trade cumulus clouds over the western Atlantic Ocean during EUREC4A‐ATOMIC. Stable water isotope ratios were sampled by isotope analyzers aboard the ship *Ronald H*. *Brown* (Bailey et al., 2023; Quinn et al., 2021). The ship sampled near‐surface air in and around a cold pool on 10 February 2020. These observations are complimented by microphysical and isotope measurements at cloud base by the WP‐3D aircraft flying nearby on 9 February 2020 (Bailey et al., 2023; Pincus et al., 2021). We use the observations from these 2 days to evaluate the sensitivity of sub‐cloud rain evaporation to microphysics, kinetic molecular diffusion, and the subcloud environment.

**Figure 1:**
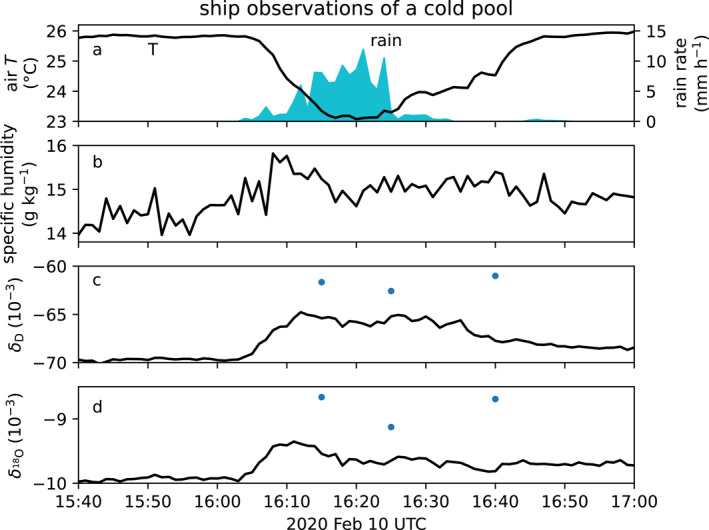
Ship time series containing a cold pool from 10 February 2020 15:40–17:00 UTC. (a) Air temperature (°C), rain rate (cyan shaded, mm $h^{-1}$), and (b) specific humidity (g ${\text{kg}}^{-1}$) at 17 m. (c) δ deuterium $\left(\text{‰}=10^{-3}\right)$ and (d) δ oxygen‐18 sampled at 18 m. Rain liquid was collected promptly during rain showers at 16:15, 16:25, and 16:40, and subsequently analyzed. Blue dots (c, d) show the vapor isotope ratio $\delta_{v}=\alpha_{e}\left(\delta_{L}+1\right)-1$ in equilibrium with the rain with isotope equilibrium coefficient $\alpha_{e}<1$ computed at T = 295 K.

Figure 1 shows the time series of a cold pool observed aboard the ship at 1‐min resolution on 10 February 2020, around 16:00 UTC at 14.3°N and 55.3°W. With air temperature cooling by 2.5°C, this cold pool is among the strongest events observed on the ship while isotope measurements were available (Quiñones Meléndez et al., 2022). Rain fell at the ship during the temperature front and minimum, suggesting the cold air and slight moistening of the specific humidity (Figure 1b) were caused by the evaporation of hydrometeors. Water vapor deuterium and oxygen‐18 isotope ratios $\delta_{D}$ and 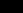 (defined in Sharp (2017)) are enriched in the cold pool compared to the background subcloud vapor measured before the cold pool (Figures 1c and 1d). Three rain samples were promptly collected in rain showers at 16:15, 16:25, and 16:40 UTC, and later analyzed to have isotope concentrations $\delta_{D}=$ 15.5‰, 14.5‰, and 16.2‰ and 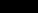 0.17‰, 0.87‰, and 0.4‰ $\left(\text{‰}\equiv 10^{-3}\right)$. We develop a case from these collocated observations of rain, temperature, and enriched isotope ratios for simulating the effects of evaporating hydrometeors.

### 2.1 Rain Evaporation Cases

A similar cloud regime was sampled differently on two consecutive days. From these observations, we construct two similar cases for our model of subcloud rain evaporation, representing rain Atlantic trade cumulus clouds. The aircraft measured the water vapor isotope ratios at cloud base and below cloud, and the rain DSD on 9 February 2020, 02:00 UTC. The near‐surface temperature and vapor isotope ratio is the mean when it was not raining, of measurements from near‐surface aircraft legs. The ship sampled the subcloud isotope ratio near the surface and collected rain (which was later analyzed) on 10 February 2020. For both cases we assume the subcloud air is adiabatically stratified and uniformly has the specific humidity and isotope ratio of the surface. The humidity and isotopic conditions for both cases are listed in Table 1.

**Table 1:** Initial Conditions at Cloud Base (Saturated) and Subcloud Vapor Conditions for Case Sarkar23 (Sarkar et al., 2023) and Case CP (10 February 2020, 16:00 UTC)

	z (m)	T (K)	$q_{v}$ (g ${\text{kg}}^{-1}$)	$\delta_{\text{D,liq}}\;\left(\text{‰}\right)$	$\delta_{\text{D,vap}}$	$\delta_{18\text{O,liq}}$	$\delta_{18\text{O,vap}}$
Case Sarkar23 (9 February 2020, 02:00 UTC)							
Cloud base vapor	700	290.0	12.9	8.68	−73	−1.75	−11.7
Subcloud vapor	0	296.8	12.9	4.54	−70	−1.14	−10.5
Rain liquid modeled	0	–	–	15.8	−59.6	2.03	−7.36
Case CP (10 February 2020, 16:00 UTC)							
Cloud base vapor	700	290.0	12.9	8.7	−73	−1.75	−11.7
Subcloud vapor	0	296.8	12.9	4.1	−70.3	−1.02	−10.4
Rain liquid modeled	0	–	–	15.4	−60.2	2.14	−7.24
Rain liquid observed	0	–	–	15.4	−60.0	0.7	−8.7

*Note*. The rain equilibrium coefficient $\alpha_{\text{eq}}\left(T\right)$ is calculated at the air temperature.

#### 2.1.1 Drop Size Distribution

**Figure 2:**
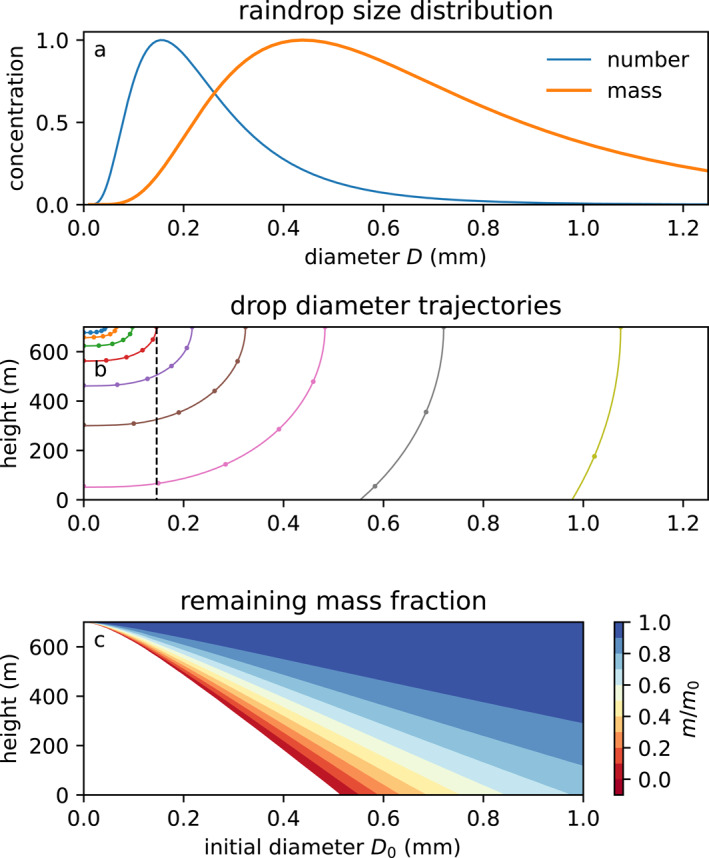
(a) The lognormal drop number N(D) and mass size distributions (Section 2.1.1). (b) Traces of individual diameters D from initial cloud‐base (z = 700 m) diameters $D_{0}$ by numerical stepping of the quadratic Equation 5. (c) Mass fraction remaining as a function of height and initial diameter. The dashed line shows D = 0.14 mm where Re ≈ 5, for which drops with smaller diameters rapidly vanish in small displacements.

The initial rain DSD is the lognormal distribution,

(1) $$N\left(D_{0}\right)=N_{0}/\left(D_{0}\sqrt{2\pi \sigma^{2}}\right)\exp\left(-{\left(\log\left(D_{0}\right)-\mu \right)}^{2}/\left(2\sigma^{2}\right)\right),$$

observed from aircraft on 9 February 2020, 02:00 UTC, in Atlantic trade wind shallow cumulus clouds in EUREC4A‐ATOMIC (Sarkar et al., 2023). The drop number concentration $N_{0}=$ 500 $m^{-3}$, the lognormal width is σ=0.35, and the lognormal mode $\mu =\log\left(D_{g}\right)$ is the log of the geometric mean diameter $D_{g}=0.22$ mm (Figure 2a), equivalent to the DSD of Sarkar et al. (2023), but with alternate notation. This DSD had the highest rain rate and most rain evaporation of any of the 22 aircraft legs.

Our model simulates the evolution of a single drop. We will explicitly predict the changing diameter of 161 individual drops representing the DSD, initially geometrically spaced with diameters $D_{0}$ between $11\times 10^{-6}$ m and 33 mm. This set of drops resolves the tails of the DSD.

#### 2.1.2 Case Sarkar23

We construct the first case, adopting the initial and environmental conditions of the 9 February, 02:00 UTC case of Sarkar et al. (2023) (called Sarkar23, Table 1). Drops are released from cloud base at 700 m and fall through a layer of uniform specific humidity $q_{v}=$ 12.9 g ${\text{kg}}^{-1}$, which is the saturation specific humidity at cloud base. Because vapor and liquid are in thermodynamic equilibrium (saturation) in clouds, the isotope ratio of the initial drop liquid $\delta_{\text{D,liq0}}=8.68\;\text{‰}$ is taken to be in isotopic equilibrium with the vapor $\left(\delta_{\text{D,vap0}}=-73\;\text{‰}\right)$ measured by the aircraft at cloud base. Temperature increases adiabatically downward, so environmental relative humidity is saturated at cloud base and 0.69 at the surface. The saturation fraction over the cooler wet bulb temperature of the drop (Appendix B) is h=0.89 at the surface.

#### 2.1.3 Case CP

We define another case for the cold pool (case CP). Except for the subcloud vapor, all other initial conditions and parameters are the same as case Sarkar23. CP uses the same initial rain liquid and DSD from Sarkar23, but modifies the subcloud layer water vapor slightly to match the background surface vapor of $\delta_{D,17m}=-69.7\;\text{‰}$ observed by the ship isotope analyzer before the arrival of the cold pool (Figure 1).

Humidity and isotopes are taken to be mixed by subcloud layer‐scale eddies above the surface layer. The height of the isotope analyzer inlet (18 m) is within the surface layer, so it will be more enriched than most of the subcloud layer due to gradients near the ocean (e.g., Thurnherr et al., 2020). We adjust humidity and isotopes by integrating the gradients through the constant‐flux surface layer (Garratt, 1992) and average the result for 150–700 m. This gives a subcloud deuterium isotope ratio of $\delta_{D,subcloud}=-70.3\;\text{‰}$, which is very close to Sarkar23's $\delta_{D,subcloud}=-70\;\text{‰}$ (Table 1). We computed the isotope ratio of the liquid in equilibrium with the vapor. CP rain liquid was collected and analyzed, and the isotope ratio of vapor in equilibrium with it was computed.

## 3 Model

Following Best (1952; and, e.g., Abraham, 1962; Li & Srivastava, 2001), the prognostic drop evaporation model evaluates the change of the square of the drop diameter as a function of the temperature and humidity of the environment. The model parameterizes diffusive kinetic effects that depend on empirical drop ventilation factors, the hydrometeor fall speed (Graf et al., 2019), and a vertically varying environment. We obtain analytical functions for drop diameter and height below cloud in Section 3.1. Isotope ratios are calculated from the drop diameters with the Craig and Gordon (1965) model in Section 3.2. A new parameterization for the kinetic effect of differential diffusion is developed in Section 3.3.

### 3.1 Drop Diameter and Mass

Sarkar et al. (2023) integrates prognostic equations for the mass, temperature, and isotope ratios of liquid water drops evaporating as they fall from the cloud base to the surface for several drop size distributions and environmental profiles observed from the NOAA WP‐3 aircraft in EUREC4A‐ATOMIC (Bailey et al., 2023; Pincus et al., 2021). We simulate the 9 February 2020 case, approximating the DSD by the lognormal distribution of Sarkar et al. (2023; Section 2.1.1).

The Lagrangian prognostic equations for a single drop are transformed from time derivatives to vertical derivatives by dividing by the fall speed $U_{\text{fall}}=-dz/dt>0$. The equation for drop diameter D evaporating with respect to height z into surrounding air with temperature $T_{a}$ and vapor density ${\hat{\rho }}_{va}$ is (Graf et al., 2019; Salamalikis et al., 2016),

(2) $$\frac{dD}{dz}=\frac{4f_{v}K_{va}}{DU_{\text{fall}}{\hat{\rho }}_{l}R_{v}}\left(\frac{e_{s}\left(T_{r}\right)}{T_{r}}-RH\frac{e_{s}\left(T_{a}\right)}{T_{a}}\right)=\frac{4f_{v}K_{va}}{DU_{\text{fall}}{\hat{\rho }}_{l}}\left(\Delta {\hat{\rho }}_{v}\right),$$

where $f_{v}$ is the ventilation factor (Stewart, 1975), $K_{va}$ is the kinematic diffusivity of vapor in air, $U_{\text{fall}}$ is the fall speed of the drop, ${\hat{\rho }}_{l}$ is the liquid water density, $R_{v}$ is the specific gas constant for water vapor, $T_{r}$ is the drop surface temperature, $e_{s}\left(T\right)$ is the saturation vapor pressure, RH is the ambient relative humidity, and $\Delta {\hat{\rho }}_{v}={\hat{\rho }}_{vr}-{\hat{\rho }}_{va}>0$ is the vapor density difference between the ambient air and the drop surface. In this equation, dD/dz>0 because the drops shrink as they fall.

We write Equation 2 in terms of the uniform specific humidity, assuming a well‐mixed subcloud layer and adiabatic temperature below cloud. The drop temperature approaches and is taken to equal the wet bulb temperature (Appendix B). Linearizing the saturation specific humidity $q_{s}\left(T\right)$ and the wet‐bulb temperature lapse rate $\Gamma_{w}$ about a mean temperature $\tilde{T}$ yields

$$\frac{dD}{dz}=\frac{4f_{v}K_{va}}{DU_{\text{fall}}{\hat{\rho }}_{l}}{\left(\frac{\partial q_{s}}{\partial T}\right)}_{\tilde{T}}\Gamma_{w}z^{\prime },$$

where $z^{\prime }$ is the displacement from cloud base. Then, the drop evaporation equation is divided into one factor that depends on D on the left hand side, and another that depends on the vertical displacement from cloud base on the right hand side:

(3) $$-a\left(D\right)D\;dD=z^{\prime }\;dz^{\prime },$$

with the function of diameter

(4) $$a\left(D\right)=\frac{{\hat{\rho }}_{l}}{4K_{va}\Gamma_{w}{\left(\partial q_{s}/\partial T\right)}_{\tilde{T}}}\frac{U_{\text{fall}}}{f_{v}}.$$

The factor a is nearly a constant, as the quotient $U_{\text{fall}}/f_{v}$ is a slowly varying function of diameter. For drops larger than 0.5 mm, $U_{\text{fall}}/f_{v}\approx 0.9$ m $s^{-1}$. At smaller diameters, the fall velocity vanishes faster than the ventilation factor, and a(D) (Equation 4) becomes very small.

Assuming that a(D) is constant, solutions of Equation 3 describe elliptical arcs centered at $D=0,z^{\prime }=0$ (Figure 2) from initial conditions $D_{0}$ and $z_{0}^{\prime }$:

(5) $$-a\left(D^{2}-D_{0}^{2}\right)=z^{\prime 2}-{z^{\prime }}_{0}^{2}.$$

Best (1952) likewise encapsulates the effect of the environment, mainly the saturation deficit, in a local “evaporation radius” K that affects the change in diameter of the drop $D^{2}-D_{0}^{2}=4K^{2}$. The drop diameter‐displacement curves are elliptical because saturation deficit increases linearly with distance from cloud base. Small departures from the linear dependence on height, such as fall speed and ventilation, are contained in a. Numerical solutions of Equation 5 show the curves of radius versus height are nearly ellipses (Figure 2b, Figure 1 of Abraham et al. (1972)), but flatten out as drops vanish and their fall speed goes to zero. We explicitly simulate individual diameters of the DSD using Equation 5 to resolve each drop's behavior as it completely evaporates.

*Drop vanishing*. The function a(D) varies little for D>0.4 mm. But in the Stokes (1856) viscous drag regime, when drops get smaller (Reynolds number Re < 5), the fall speed $U_{\text{fall}}\propto D^{2}$ and a(D) vanish over small vertical displacements. This squashing of the displacement near vanishing due to the dependence on $U_{\text{fall}}/f_{v}$, can be parameterized by the function

(6) $$a\approx a_{0}\frac{D^{2}}{D^{2}+b^{2}},$$

with parameters $a_{0}=2.1\times 10^{12}$ and b=0.2 mm. This approximation estimates the displacement at which drops vanish. The factor $D^{2}/\left(D^{2}+b^{2}\right)$ approaches unity in the falling regime and shrinks as the drops vanish. Solution and surface tension effects are important for very small droplets and concentrated solutions (e.g., haze and sea spray; Andreas, 1995), but these effects are neglected here.

The quasi‐elliptical trajectories of drop diameter versus displacement fallen can be evaluated in midpoint prediction‐correction steps of drop diameter using Equation 5. It is accurate to evaluate in a few (e.g., $n_{k}=5$) cosine‐spaced steps $D_{k}=D_{0}\;\cos\left(\pi k/\left(2n_{k}\right)\right)$ (Figure 2b, circles). However, to find the resulting size of all drops at each height, we use 1 m vertical resolution (Figure 2b, lines). The 5‐step evaluation agrees with the high‐resolution solution because the slope a(D) varies slowly with respect to diameter. A single step $\left(n_{k}=1\right)$ overestimates the displacement that small drops fall because of the curvature of a(D) for $D\tilde{<}b\approx 0.2$ mm. The approximation for a (Equation 6) accurately predicts the displacements at which drops completely evaporate. Drops initially smaller than $D_{0crit}=$ 0.51 mm will be shown to evaporate completely within 700 m below cloud base.

### 3.2 Isotope Exchange

The change in the isotope ratio of a single liquid drop is calculated from the mass fraction $f={\left(D/D_{0}\right)}^{3}$ of drop liquid remaining, using the Craig and Gordon (1965) and Stewart (1975) model. Single‐drop results are then integrated over the DSD.

Craig and Gordon (1965) and Stewart (1975) assume finite diffusion between the equilibrium vapor over the drop and the surrounding air. The drop isotope ratio $R_{L}$ is predicted by the following:

(7) $$R_{L}-cR_{\mathrm{air}}=\left(R_{L0}-cR_{\mathrm{air}}\right)f^{A},$$

where f is the mass fraction of the drop remaining, $R_{\mathrm{air}}$ is the isotope ratio of the vapor in the air, and $R_{L0}$ is the isotope ratio of the original liquid. The exponent is

$$A=\frac{\rho }{\rho_{i}}\frac{\alpha_{e}}{1-h}-1,$$

where $\rho /\rho_{i}$ is the ratio of the resistance of total water evaporation over the resistance of evaporation of isotope i, $\alpha_{e}=R_{V}/R_{L}<1$ is the equilibrium fractionation factor of the isotope ratio of vapor over that of liquid and $h=q/q_{s}$ is the saturation fraction over the liquid surface. The coefficient c is given as follows:

(8) $$c=\frac{h}{\alpha_{e}-\left(1-h\right)\left(\rho_{i}/\rho \right)}.$$

Including exchange with environmental vapor and diffusive effects replaces $\alpha_{e}$ in the Rayleigh process with $\alpha_{e}\left(\rho /\rho_{i}\right)/\left(1-h\right)$. Rayleigh evaporation assumes vapor at equilibrium over the drop irreversibly leaves the drop as it evaporates. The isotope ratio of the liquid is then distilled (Rayleigh):

$$R_{L}=R_{L0}f^{\alpha_{e}-1}.$$

The initial condition for Equation 7 is f=1. As the fraction of mass remaining vanishes, f→0, the final isotope ratio approaches

(9) $$R_{Lend}=cR_{\mathrm{air}}.$$

For saturation fraction h=0, $cR_{\mathrm{air}}$ drops out, giving $R_{L}=R_{L0}f^{\alpha_{e}\rho /\rho_{i}-1},$ which is like the Rayleigh solution, except for $\rho /\rho_{i}$ in the exponent.

### 3.3 Kinetic Effect of Diffusion From Evaporating Drops

Craig and Gordon (1965) and Stewart (1975) parameterize evaporation E=[1−h(∞)]/ρ as the ratio of the saturation deficit of the surroundings 1−h(∞) and a resistance ρ. The resistance is inversely related to the vapor diffusivity. A spherical drop evaporates by down‐gradient diffusion of water vapor, parameterized by a series of molecular diffusivity $K_{m}$ and eddy diffusivity $K_{e}$. The net drop evaporation, nondimensionalized by the surface saturation vapor density ${\hat{\rho }}_{vsat}$, reduces the mass m of the drop, $E=-{\left({\hat{\rho }}_{vsat}\right)}^{-1}\left(dm/dt\right)$. Evaporation is a product of the diffusivity K and the radial derivative of saturation fraction $h={\hat{\rho }}_{v}/{\hat{\rho }}_{vsat}$,

$$E=-4\pi r^{2}K\frac{dh}{dr}.$$

Assuming spherical symmetry, steady state, and water continuity, we integrate the saturation fraction h(a)=1 from the drop surface at radius a to arbitrary radius r. Then the saturation fraction h as a function of radius is given by:

(10) $$1-h\left(r\right)=\int_{r}^{a}dh=\frac{E}{4\pi }\int_{r}^{a}\frac{1}{K}\frac{dr^{\prime }}{r^{\prime 2}}=\frac{E}{4\pi }\frac{1}{K}\left(\frac{1}{a}-\frac{1}{r}\right).$$

Now we parameterize the diffusivity K. Molecular diffusivity $K_{m}$ varies for different isotopes. The eddy diffusivity $K_{e}$ depends on the flow around the drop but is assumed to be the same for all isotopes. The diffusivity ratio of a specific isotope to the abundant water, $K_{mi}/K_{m}$, is 0.9755 for deuterium and 0.9723 for oxygen‐18 (Merlivat, 1978).

We model the diffusion through concentric spheres, with molecular diffusivity $K_{m}$ from the drop radius a through a layer of thickness l, and with eddy diffusivity $K_{e}$ outside radius a+l. Integrating to different radii gives $\rho =\rho_{m}+\rho_{e}$ with

$$\rho_{m}=\frac{1}{4\pi K_{m}a}\;\frac{l}{a+l}\;\;for\;\;r=\left[a,a+l\right],$$

and

$$\rho_{e}=\frac{1}{4\pi K_{e}}\;\frac{1}{a+l}\;\;for\;\;r=\left[a+l,\infty \right).$$

The ratio of resistances $\rho_{i}/\rho =1+n\left(K_{m}/K_{mi}-1\right)$ of flux of the rare isotopologue i to the flux of abundant water vapor depends on the drop size through the ratio of molecular to total (molecular + eddy) resistance $n=\rho_{m}/\rho$. Stewart (1975) and Kinzer and Gunn (1951) found n=0.58 for drops in the range 1.4–2.8 mm diameter. Our spherical diffusion model is solved to yield the ratio of molecular to total resistance as:

(11) $$n={\left(1+\frac{K_{m}}{K_{e}}\frac{a}{l}\right)}^{-1},$$

which depends on the ratio a/l of the drop size to the viscous‐diffusive length scale l.

**Figure 3:**
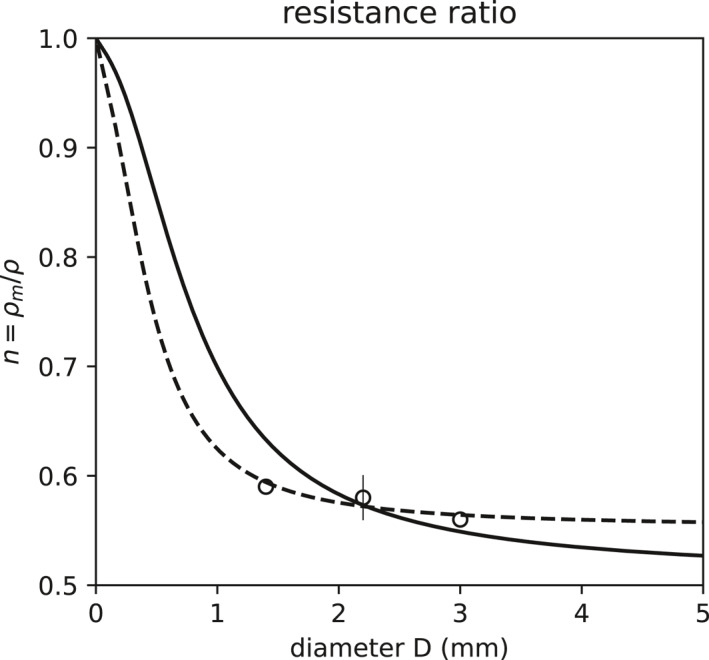
Resistance ratio $n=\rho_{m}/\rho$ parameterization (Equation 11, solid) and the ad hoc empirical parameterization $\tilde{n}$ (Equation 12, dashed), with lab experiment results of Kinzer and Gunn (1951, circles) and Stewart (1975, circle and whisker).

Diffusion through this spherical geometry predicts vanishingly small drops approach n=1. Flow separates from large drops and the diffusion loses its spherical geometry. Vapor diffuses away from small drops through concentric spheres, as above. Choosing total layer thickness

$$l=l_{\nu }+l_{e},$$

with $l_{\nu }=\nu /U$ and $l_{e}=\left(K_{m}/K_{e}\right)a$, matches the rough limit n=1/2 (Brutsaert, 1965, 1975; Merlivat & Jouzel, 1979) for large drops. The relative velocity scale U is essentially the fall speed, at which drag and gravity balance. (Appendix A, shows how to augment U slightly to include turbulent inertial accelerations.) The parameterization matches the experimental results for drops of diameter 1.4, 2.1, and 3 mm from Kinzer and Gunn (1951) and Stewart (1975) (Equation 11, Figure 3 solid) when setting the molecular to eddy vapor diffusivity ratio to $K_{m}/K_{e}=6\times 10^{-3}$. An ad hoc alternative parameterization matching the experiments, that asymptotes instead to n=0.55 for large drops, is given as follows:

(12) $$\tilde{n}=0.55+0.45{\left(1+0.04a/l_{\nu }\right)}^{-1}$$

(Figure 3 dashed). Hereafter, we use the more physical parameterization (Equation 11).

Models such as Lee and Fung (2008), Graf et al. (2019), and Sarkar et al. (2023) include the effect of differential diffusion through empirical ventilation factors that depend on the diffusivity of each isotopologue species. Our approach follows Stewart (1975), resolving kinetic effects by explicitly parameterizing the flow‐ and geometry‐dependence of the ratio of laminar and turbulent resistances. Where Stewart had determined this experimentally for 1–3 mm drops, we parameterize the effect as a function of the size of any falling drop. This explicit treatment of diffusivity predicts kinetic effects on the isotopes of drops even as they vanish.

## 4 Isotope Simulation Results

Results from modeling the evaporation of drop mass for drops of different sizes with Equation 3 and evaluating the isotope ratio $R_{L}$ of each drop using the resulting mass fraction f and Equation 7 are presented for three types of experiments. First, we present simulations from case CP representative of a cold pool near rain observations (Section 2) and compare it to vapor observed at the surface. Second, we show sensitivity experiments to the width and geometric mean diameter of the DSD, tests with a monodisperse DSD, and a test with constant relative humidity below cloud. We compare the simulations to results from simpler mixing and Rayleigh distillation models. Third, we demonstrate a set of idealized physics experiments, each with different physical effects selectively zeroed out by artificially setting combinations of the difference between the isotope ratio of the drop and the air to zero, the equilibrium fractionation coefficient to unity, and/or the diffusive fractionation coefficient to unity.

### 4.1 Simulations From Trade Cumulus Case CP

**Figure 4:**
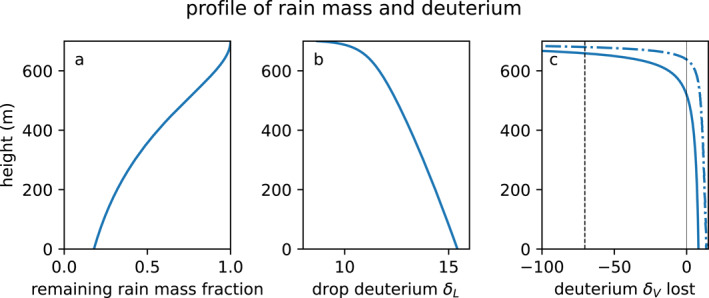
(a) Mass fraction of total liquid remaining versus height. (b) Mean deuterium δ (‰) of all the liquid remaining. (c) Deuterium δ of drop evaporation at each height (dot‐dashed) and cumulative evaporated vapor (solid). Black dashed (c) shows the isotope ratio of the surrounding vapor.

We first evaluate the isotope ratio $R_{L}$ of each drop using the mass fraction f and Equation 7 for the cold pool case (CP, Table 1). The total mass fraction and deuterium composition of the remaining DSD and the vapor lost is shown in Figure 4. Figure 4c shows the deuterium composition of the immediately evaporated vapor (dot‐dashed) and the cumulative vapor (solid), compared to the surrounding subcloud vapor (black dashed) in delta notation $\delta =R/R_{s}-1$. The cumulative vapor below 650 m is enriched compared to the surroundings. It is enriched to δ=0‰ below 500 m. A small fraction of this enriched vapor could explain the enrichment observed in cold pools in Figure 1. The immediately evaporated vapor at each level is yet more enriched than the previously evaporated vapor.

**Figure 5:**
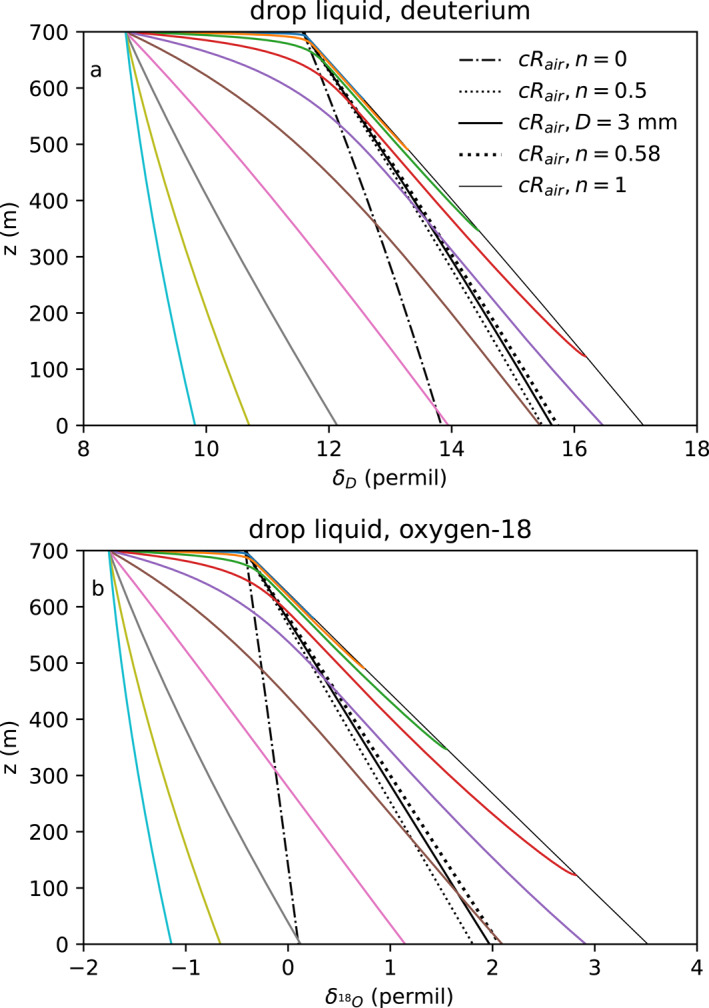
(a) Deuterium and (b) oxygen‐18 isotope trajectories (colored lines) for drop with initial diameter $D_{0}$ of 0.13 (blue, top), 0.20, 0.29, 0.44, 0.65, 0.97, 1.45, 2.2, 3.2, and 4.8 (cyan, lowest) mm. Our parameterization for the end point $R_{\text{end}}=cR_{\text{air}}$ for vanishing drops with n→1 (thin black). No kinetic effect $\rho_{i}/\rho =1,\;n=0$, equilibrium fractionation only (dot‐dashed black). $R_{\text{end}}$ for n=0.5 (thin dotted black) and n=0.58 (Stewart, 1975, dotted black), which matches extrapolating our model for diffusion of large (3 mm) drops (black solid).

The isotope ratio is shown for 10 drops with diameters of 0.13–4.8 mm (Figure 5). The initial isotope ratio is 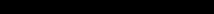 $\left(\text{‰}\equiv 10^{-3}\right)$. As they fall, drops evaporate fractionally more of their lighter $H_{2}$O isotopologues and enrich monotonically. Curves that strike z=0 reach the surface. The largest drop shown (4.8 mm; cyan left) nearly reaches $\delta_{D}=10\text{‰}$ at the surface. Smaller drops fall slower, evaporate more and become more enriched over a shorter distance. The smallest drop shown (blue, top) quickly reaches equilibrium with the surrounding vapor, before evaporating completely between 500 and 600 m. The largest drop shown here that evaporates completely (0.44 mm, red) enriches to $\delta_{D}=16\;\text{‰}$, where it vanishes around 150 m. The enrichment curves for water containing deuterium or oxygen‐18 isotopes appear quite similar, differing mostly due to the difference between isotope ratio of the initial drop and the equilibrium liquid isotope ratio of the air (at the intersection of the black lines at cloud base). The initial difference is stronger, compared to its kinetic effect, for deuterium than for oxygen‐18.

All drops enrich by exchange with the surrounding vapor, from the drop initial condition, toward equilibrium with the vapor. The equilibrium liquid isotope ratios of the air at h=1 (shown at z = 700 m) are $\delta_{\text{D,end}}=11.6\text{‰}$ and 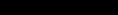 for the subcloud air vapor isotope ratios of $\delta_{\text{D,air}}=-70.3\text{‰}$ and 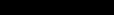. Large drops fall through the layer exchanging only slightly, while small drops exchange quickly toward equilibrium with the enriched ambient vapor. Lee and Fung (2008) use the rate at which drops reach isotopic equilibrium with relatively enriched environmental vapor as a possible explanation for the amount effect.

In addition to this exchange and equilibration, kinetic effects further enrich the drop isotope ratio toward a kinetically enhanced end point (thin black line, Figure 5) at n=1 for vanishingly small drops D→0. This end point is even more enriched at lower relative humidity. The heavier water isotopologues diffuse away from the drops more slowly than ${H_{2}}^{16}$O, enriching the drops. The black lines show the end point isotope ratio of liquid drops predicted by different parameterizations of the kinetic fractionation (i.e., different values of n). At cloud base (700 m, h=1) all endpoint curves intersect the vertex of equilibrium with the environmental vapor. Drops approach their end point isotope ratio $R_{\text{end}}=cR_{\text{air}}$ (thin black line) predicted by Equation 8. The kinetic effect of differential diffusion is stronger as n approaches unity for small drops. The isotope ratio $R_{\text{air}}h/\left[\alpha_{e}-\left(1-h\right)\right]$ (black dot‐dashed) is the end point for the artificial case of n=0, which would be obtained were the rare isotope vapor diffusivity equal the abundant isotope diffusivity.

Estimates of $R_{\text{end}}$, using our n parameterization (Equation 11) to model $\rho_{i}/\rho$ for large (3 mm) drops (black solid), agree with results for n=0.58 (black dashed), found for lab experiments on drops larger than 1 mm diameter (Stewart, 1975). The n=0.58 end point underestimates kinetic enrichment as drops shrink. Even drops as small as 0.13 mm fall more than 100 m with their isotope ratio greater than the endpoint $R_{\text{end}}$ predicted by n=0.58. The enrichment of $\delta_{\text{end}}$ is approximately proportional to n. Our parameterization for n, which approaches n=1 and $\left(\rho_{i}/\rho =K_{m}/K_{mi}\right)$ for small drops has about 42% more kinetic enrichment than taking constant n=0.58.

Though the mass of vanishing drops is small, rain transports enriched liquid downward, leaving relatively depleted vapor near cloud base. Small drops and virga (raindrops that evaporate completely before reaching the surface) experience the strongest enrichment, and their complete evaporation moves very enriched vapor downward in the subcloud layer. Virga that evaporates just before reaching the surface is enriched, by +7‰ for deuterium and by +5‰ for oxygen‐18, with kinetic effects of +5.8‰ and +3.5‰, respectively. This evaporation of enriched hydrometeors (both large and small) explains enrichment of vapor observed in evaporatively cooled cold pools in EUREC4A‐ATOMIC (Section 2).

#### 4.1.1 Exchange and Kinetic Evaporation

The ratio of the change of deuterium to oxygen‐18 isotope ratio is often analyzed in terms of deuterium excess (DXS) DXS 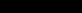. Isotope ratios $\delta_{D}$ and 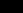 of precipitation vary, on average, along the global meteoric water line (GMWL). The GMWL has nearly constant DXS, reflecting the dominance of equilibrium fractionation of precipitation. Departures from the GMWL represent local differences in the ratio of equilibrium fractionation, as well as nonequilibrium kinetic fractionation processes.

**Figure 6:**
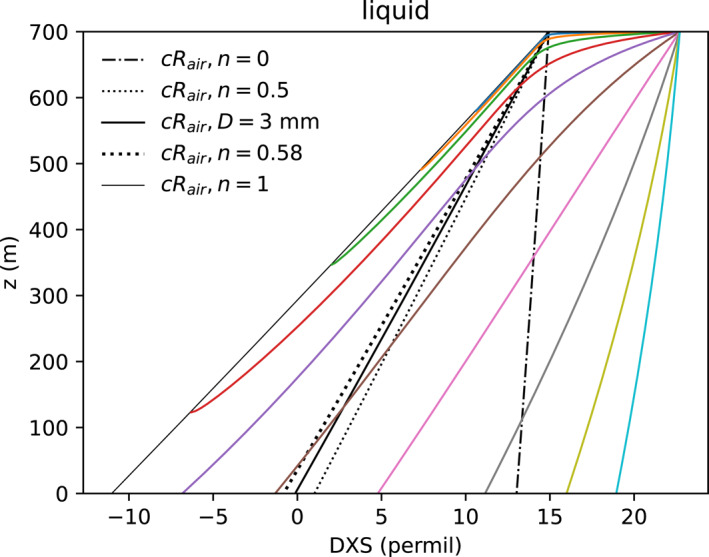
Deuterium excess 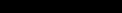 profiles as in Figure 5.

**Figure 7:**
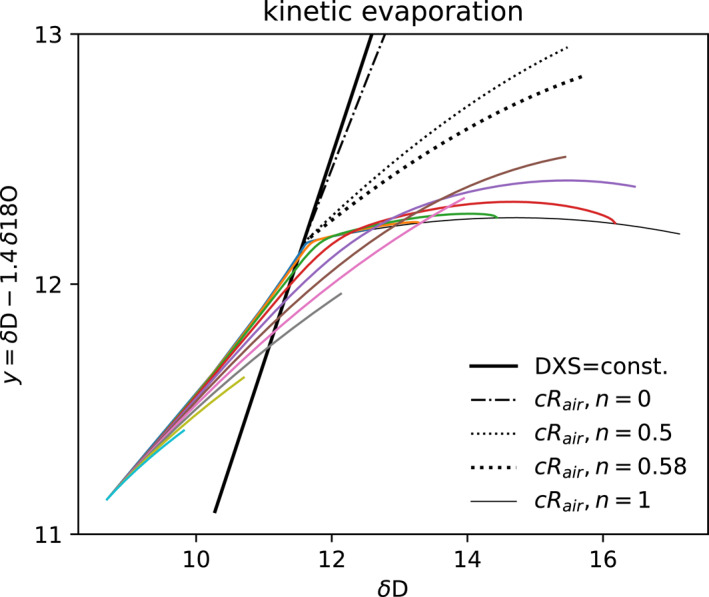
Isotopic trajectory of drops exchanging with subcloud vapor (intersection of black lines) and then undergoing the kinetic evaporation process. The vertical axis 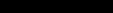 distinguishes these effects. The thick solid black line represents constant deuterium excess (DXS). DXS of the evaporating drops decreases (with lower DXS to the right). The steady state end points are indicated by black dashed and thin lines as in Figures 5 and 6. Vanishing drops tend toward nearly constant y.

Rain evaporation does not follow a meteoric water line. Rather, exchange and kinetic effects decrease the DXS of drops as they fall (moving to the right in Figures 6 and 7). Diffusion of HDO vapor is inhibited less, relative to the abundant $H_{2}$O, than is the vapor diffusion of $H_{2}^{18}$O as drops evaporate. Thus for subcloud evaporation, the drop liquid ratio of HDO increases relatively less than predicted by equilibrium evaporation.

Differences in the initial DXS of the rain and subcloud boundary layer set the drops up for decreasing their DXS by exchange. DXS of the subcloud vapor (intersecting black lines, Figure 7) is 12.3‰, 10‰ lower than the initial drop condition from the equilibrium cloud (DXS = 22.7‰). We presume the initial DXS of rain is high by the prior removal of low‐DXS precipitation from the cloud.

Small drops are found to approach 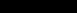 as kinetic effects dominate their fractionation. Thus Figure 7 uses an unconventional isotopic coordinate 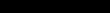 to highlight the difference between exchange and kinetic evaporation. Kinetic fractionation with n=1 has approximately constant y. The subcloud vapor has the DXS of the thick black line, and $\delta_{D}$ and y of its intersection with the equilibrium and kinetic process (black dashed) lines. The slope of the local equilibrium evaporation process (n=0, dot‐dashed) has approximately constant DXS. The drops start initially with higher DXS (left). DXS decreases (rightward) toward the DXS of the subcloud vapor by exchange. The DXS of most drops crosses the subcloud vapor DXS and continues to decrease by kinetic enrichment. The drops approach the kinetic limit defined by $\delta_{\text{end}}=cR_{\text{air}}/R_{s}-1$ for n=1 with a nearly constant value of y = 12.1‰ (thin black line).

#### 4.1.2 Vapor Lost by Hydrometeors

**Figure 8:**
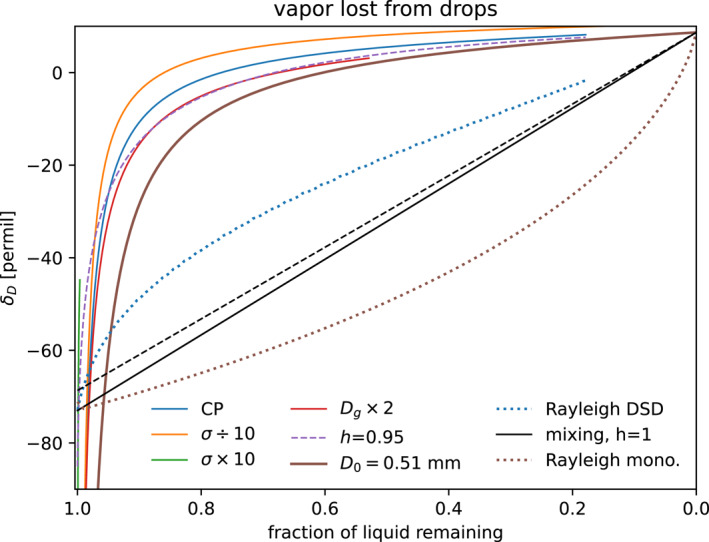
Total isotope δ of vapor evaporated from raindrops, as a function of fraction f of rain mass remaining for case CP (blue solid). Evaporation of drop mass is modeled for drops of different sizes with Equation 3, and isotopes are simulated by the Craig and Gordon isotope evaporation model with diameter‐dependent kinetic effect (Sections 3.2 and 3.3). Sensitivity experiments have drop size distribution (DSD) with 10× narrower (orange) or wider (green) lognormal width σ, 2× larger geometric mean diameter $D_{g}$ (red), have surroundings with fixed T and h=0.95 representative of 400 m (purple dashed), or represent the single drop of initial diameter of 0.51 mm (brown solid). Mixtures of $f=f_{\text{eq}}$ equilibrium vapor from negligibly evaporated drops and $1-f=f_{\text{complete}}$ vapor from completely evaporated drops of initial liquid isotope ratio (black: solid for vapor equilibrium over the drop evaluated at cloud base (700 m) and dashed for vapor equilibrium evaluated at the surface). Rayleigh evaporation of the control case DSD (blue dashed), and a monodisperse DSD (single drop, brown dashed).

The instantaneous and cumulative isotope ratio of the vapor evaporated from all hydrometeors in the DSD is shown as a function of height in Figures 4b and 4c, and as a function of the mass fraction of original hydrometeor liquid in Figure 8. The isotope ratio of the vapor lost by the drops is sensitive to the effect of kinetic fractionation in the Craig and Gordon (1965) evaporation equation (CG, Equation 7) and the DSD. CG generates concave‐down curves that enrich quickly at first, that is, at low mass fraction evaporated, and then slowly approach the isotope ratio of the original liquid $\delta_{D0}$. The CG model for a single drop, or equivalently a monodisperse DSD (brown), is still concave‐down. Thus, the shape of these curves is mostly due to the CG physical model, not the shape of the DSD.

#### 4.1.3 Rain Isotope Flux

Figure 4 shows the integrated effect of rain is to enrich rainwater quickly below cloud (because the initial rain isotopic composition was in equilibrium with somewhat more depleted cloud layer air), and then evaporate this enriched water lower in the subcloud layer. This suggests a downward flux of heavy isotopes by the rain. This up‐gradient flux acts to strengthen the observed depletion of heavy isotopes with altitude in the atmosphere.

The vertical flux of water isotopes by the rain is quantified from the model simulations:

(13) $$F_{\delta rain}=\sum_{j}M_{j}\left(\delta_{j}-\bar{\delta }\right),$$

where $M_{j}=-\left(\pi /6\right){\hat{\rho }}_{l}D_{j}^{3}U_{\text{fall},j}N_{j}$ is the mass flux of drops with isotope ratio $\delta_{j}$ and diameter $D_{j}$, with number concentration per volume, $N_{j}=N\left(D_{0,j}\right)$, given by the initial DSD, and $\bar{\delta }$ is the mean isotope concentration δ of total water, which is dominated by the subcloud vapor.

In our steady state model, the total water isotope source to the air‐rain mixture due to rain is the vertical convergence (−∂/∂z) of the rain isotope flux is as follows:

(14) $$S_{\delta rain}=-\frac{1}{{\hat{\rho }}_{v}}\frac{\partial }{\partial z}F_{\delta rain}=\frac{1}{{\hat{\rho }}_{v}}\sum_{j}\left[-\frac{\partial M_{j}}{\partial z}\left(\delta_{j}-\bar{\delta }\right)-M_{j}\frac{\partial }{\partial z}\left(\delta_{j}-\bar{\delta }\right)\right],$$

where ${\hat{\rho }}_{v}$ is the vapor density per unit of total air. The source is separated into two physically distinct parts: on the left, the effect of bulk rain evaporation, and on the right, the effect of advection of the isotopes by the rain.

**Figure 9:**
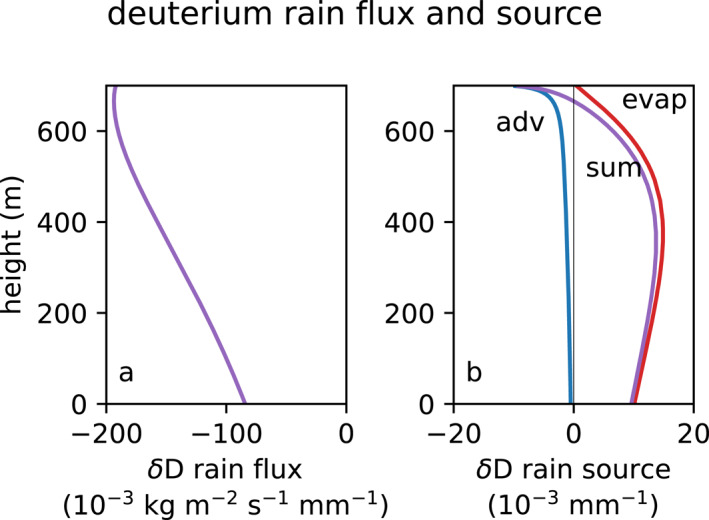
(a) Total isotope δ flux by rain. (b) Total isotope source (purple) from case CP, and its contribution from isotope advection (adv, blue), and rain mass flux divergence from evaporation (evap, red).

The mass flux, isotope flux, and source rates all scale with the rain rate (and $N_{0}$); the integrated source scales with the rain accumulation. We evaluate the rain flux of δ for the deuterium isotope for a nominal rain rate of $M_{j}/{\hat{\rho }}_{l}=1$ mm $h^{-1}$ (Figure 9a), and the integrated source for 1 mm of precipitation accumulation at the surface (Figure 9b).

The weaker term of the flux divergence is the advection of the isotope by the rain. Since the mean $\bar{\delta }$ is nearly constant, the effect of the advection term is mostly within the liquid phase. Drops enrich by evaporation and exchange, especially near cloud base, causing strongly negative $\partial \delta_{j}/\partial z$. This gradient results in negative isotope advection by the falling rain.

The stronger term is the evaporation (Figure 9b, red). Removing rain mass causes convergence of the rain mass flux. Drops are enriched compared to the subcloud vapor, explaining the large enrichment by the evaporation. The vertically averaged subcloud rain evaporation source enriches an undiluted precipitating core by +11.8‰ per mm rain. The precipitation accumulation for the cold pool on 10 February at 16:00 UTC (Figure 1a) is 1.9 mm. Evaporation from this accumulation would enrich precipitation downdraft air by 22‰. This contributes to the cold pool, whose deuterium δ is enriched by +4.9‰ (Figure 1c). Were hydrometeor evaporation the only source, dilution of the evaporative core by 3.6 times as much surrounding vapor would explain the vapor isotopes observed in the cold pool. In fact, other sources in addition to hydrometeor evaporation, such as evaporation from the ocean surface, also contribute to the near surface air in cold pools (Quiñones Meléndez et al., 2022).

### 4.2 Sensitivity Experiments

#### 4.2.1 Sensitivity to the DSD

Figure 8 presents the evolution of the isotope composition of evaporated vapor for the standard DSD ($D_{g}$ = 0.22, σ = 0.345, in blue) along with a number of sensitivity studies. Results are not sensitive to narrowing the DSD's lognormal width σ by a decade or to doubling the geometric diameter $D_{g}$. The orange curve shows a narrower distribution, with decimated lognormal width σ = 0.0345. A wider σ = 3.45 distribution barely evaporates, and almost all liquid remains at the surface (green; almost invisible at left) because much of the mass is in drops too large to evaporate. Because of the nonlinear effect of complete evaporation of small drops versus the persistence of large drops, the effect of the width on the isotopes is not monotonic. The narrower and wider distributions both evaporate less and enrich faster than the standard DSD.

Doubling $D_{g}$ to 0.44 mm (red) reduces the fraction of rain evaporated, 1−f, between cloud base and the surface, from 0.8 with the standard DSD to ∼0.5. Vapor also enriches slightly more slowly, suggesting relatively more evaporation from larger less enriched drops than from the control DSD.

A case with a single drop, equivalent to a monodisperse DSD, shows the vapor resulting from the initial drop size ($D_{0}$ = 0.51 mm, Figure 8 brown curve) that completely evaporates at the surface. Preferential and near‐complete evaporation of the smaller drops of the DSD (blue) are responsible for about +10‰ more enrichment of the cases with a DSD, compared to the case with a single drop.

#### 4.2.2 Sensitivity to the Environmental Profile

The case indicated by the dashed purple line in Figure 8 uses uniform subcloud humidity h=0.95 (representative of 400 m), to simplify the effect of the environment. The main difference is that it starts off evaporating near isotopic equilibrium, compared to the transient strongly depleted vapor right below cloud base where h is nearly unity (blue line, off scale). Away from cloud base, the uniform environment has only a small effect on the isotope ratio of the evaporated vapor, and the evaporated fraction 1−f is almost identical to the standard case.

#### 4.2.3 Comparison to Simpler Models

The simplest model for the isotope ratio of vapor is linear mixing between the initial liquid and its equilibrium vapor. Mixtures between the “first whiff” of initial equilibrium vapor, and the “final gulp” of completely evaporated drops appear between the straight black lines. The equilibrium varies slightly with h: the lower (solid) mixing line in Figure 8 represents equilibrium at cloud base h=1, and the upper dashed line represents the equilibrium at the surface h=0.89.

The Rayleigh model for a single drop with $D_{0}$ = 0.51 mm is concave up (brown dotted line in Figure 8). This single‐drop Rayleigh model performs worse than linear mixing (black lines). Accounting for the DSD by using the one from the EUREC4A‐ATOMIC observations (Section 2.1.1), the Rayleigh model is significantly improved and has a concave down shape in Figure 8 (blue dotted line), falling between linear mixing and the more physical CG solutions. The vapor exchange and kinetic fractionation effects additionally included in the CG model have a stronger effect on the results than the DSD. Evaluating CG even for a single average‐sized drop gives a considerably better result than the Rayleigh model for the whole DSD.

#### 4.2.4 Sensitivity to Isotope Ratio of the Initial Drop and Subcloud Vapor

The rain at the surface is largely equilibrated through exchange with the vapor in the subcloud air. This exchange was identified in early isotope‐enabled general circulation models as a factor for predicting local precipitation‐temperature relationships (Noone & Simmonds, 2002). Experiments varying the initial drop isotope ratio and the isotope ratio of the vapor in the subcloud air, show the isotope ratio of the surface rain (integrated by mass over the DSD) depends strongly on the isotope ratio of the vapor in the air, and slightly on the drop initial conditions. The sensitivity for deuterium anomalies is

(15) $$\delta_{\text{pcp,sfc}}^{\prime }=0.972\alpha_{e}^{-1}\delta_{\text{air}}^{\prime }+0.036\delta_{L0}^{\prime },$$

with $\delta^{\prime }=\delta -\delta {}^{\circ}$ indicating anomalies from reference conditions δ°, which are for the standard cold pool case: $\delta_{\text{pcp,sfc}}^{◦}=15.4\;\text{‰},\delta_{\text{air}}^{◦}=-70.3\;\text{‰},$ and $\delta_{L0}^{◦}=8.68\;\text{‰}$. The coefficients are constant over a wide range of observed conditions: $\delta_{\text{air}}=\left[-69,-72\right]\;\text{‰}$ and $\delta_{L0}=\left[9,12\right]\;\text{‰}$.

The sensitivity experiments demonstrate that the exchange and kinetic effects in the CG evaporation model make a significant difference to the results. The CG model is not significantly more complex than a Rayleigh model, yet it is significantly more accurate. Microphysics also makes a difference: Changing the DSD determines which drops evaporate completely, and this complete evaporation profoundly enriches the resulting vapor.

### 4.3 Idealized Physics Experiments

Idealized experiments illustrate the effects of exchange with the environmental vapor, and the evaporative distillation by equilibrium and kinetic fractionation of the isotopes between the liquid and vapor phases. The same drop sizes as in case Sarkar23 and CP are reused for all the experiments. In the first three experiments, the initial drop liquid and environmental vapor in the air are in isotopic equilibrium. The Control case has all three effects on the isotopes: exchange with the environmental vapor, distillation by equilibrium fractionation, and distillation by diffusive (kinetic) fractionation. The second case (OnlyDiff) has only environmental exchange and differential kinetic diffusion. Its equilibrium fractionation $\alpha_{e}=1$ is artificially set to unity to suppress equilibrium distillation. The third case (OnlyEq) has only environmental exchange and equilibrium distillation. Its molecular diffusivity ratio $K_{m}/K_{mi}$ is set to unity to eliminate diffusive kinetic fractionation. All the experiments have environmental exchange. Since these three cases have uniform subcloud air with $\delta_{L}^{\text{veq}}=0$, in equilibrium with the liquid drops $\left(\alpha_{e}^{-1}R_{\mathrm{air}}=R_{L}=R_{s}\right)$, environmental vapor exchange has the effect of simply relaxing back to δ=0.

**Figure 10:**
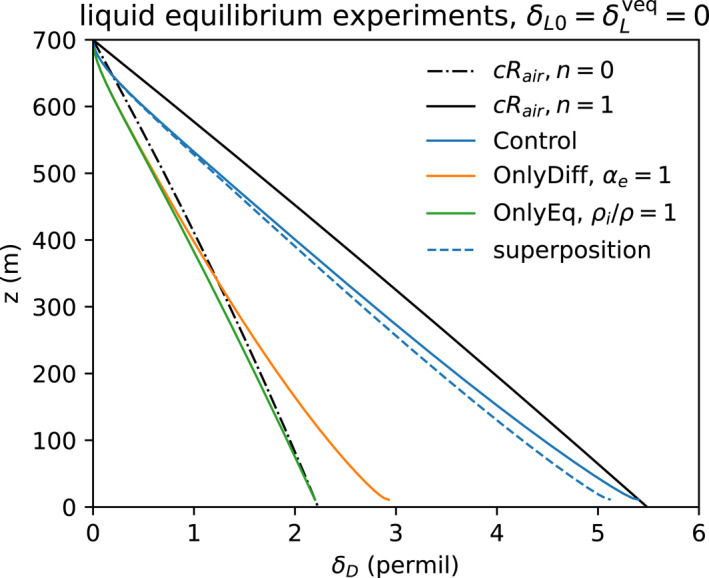
Deuterium isotope δ of drops of initial diameter $D_{0}=0.51$ mm and isotope ratio δ=0 for three experiments with air in isotopic equilibrium with the initial drop liquid isotope ratio $\delta_{L0}=\delta_{L}^{\text{veq}}=0\;\text{‰}$: Control (solid blue) includes exchange, distillation by equilibrium fractionation, and distillation by diffusive fractionation; OnlyDiff (orange) includes equilibrium and diffusion; and OnlyEq (green) includes exchange and diffusion. The dashed blue line shows the linear superposition δ sum of OnlyDiff and OnlyEq.

Trajectories for drops with initial diameter $D_{0}=$ 0.51 mm, which evaporate completely just above the surface, are shown for these four experiments in Figure 10. The black lines are the steady state end points for n=0 and 1, as in Figure 5. The steady state end point depends on relative humidity over the drop h, which goes from 1 at cloud base to 0.89 at the surface.

The Control experiment (blue, Figure 10), with all 3 effects, reaches the end point of $\delta_{D}>5\text{‰}$ defined by n=1. Differential isotope diffusive fractionation (OnlyDiff) and equilibrium fractionation (OnlyEq) mechanisms both enrich drops by similar amounts and the linear sum of these effects is only slightly less than their combined effect in the Control experiment. For diffusive fractionation without equilibrium fractionation (OnlyDiff: orange, Figure 10), δ approaches a final value of +3‰. For equilibrium without diffusive fractionation (OnlyEq: green, Figure 10), $\rho_{i}/\rho =1$ is achieved by $K_{m}/K_{mi}=1$, and isotopes increase by +2‰, reaching the end point for n=0 (black dot‐dashed). Setting n=0 also excludes differential diffusion. The end points increase downward due to decreasing relative humidity. About half of the effect of relative humidity on the end point is counteracted by reduction of $\alpha_{e}^{-1}=\alpha_{eL/V}$ with increasing temperature.

**Figure 11:**
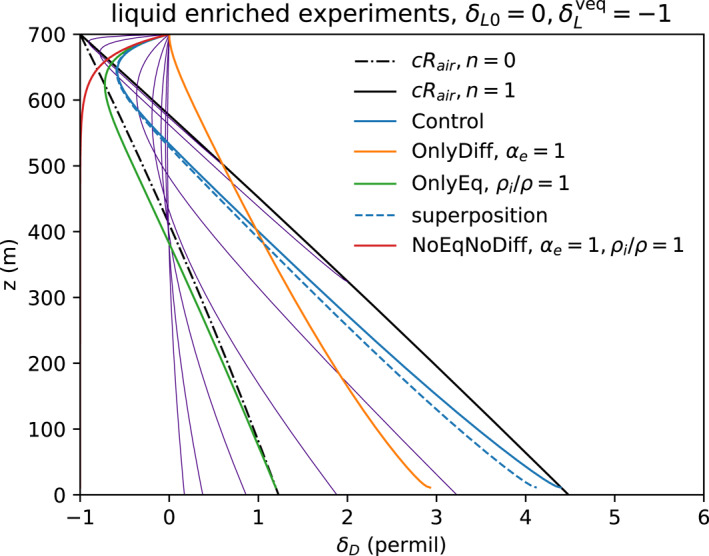
Deuterium isotope experiments Control (solid blue), OnlyDiff (orange), and OnlyEq (green) as in Figure 10 but with environmental air depleted by −1‰ compared to equilibrium over the drop. A fourth experiment, NoEqNoDiff (red), has only exchange with air. Purple lines show results for different drop sizes, with smaller drops adjusting over shorter displacements.

In Figure 11, these experiments (Control, OnlyDiff, and OnlyEq) are repeated for a depletion of the environmental air that shifts the equilibrium isotopic composition of the initial drop to $\delta_{L}^{\text{veq}}=-1\;\text{‰}$. This depleted environment is unrealistic, but it allows us to distinguish enrichment by fractionation from (in this case) depletion by exchange. The exchange process immediately acts to deplete the drops toward δ=−1 from the initial $\delta_{L0}=0$. A fourth case (NoEqNoDiff, red Figure 11) with only environmental exchange (having both equilibrium fractionation and diffusional fractionation disabled by setting to unity $\alpha_{e}=1$ and $K_{mi}/K_{m}=1$) illustrates this, relaxing to $\delta_{L}^{\text{veq}}=-1\;\text{‰}$ with a length scale of 40 m below cloud base for the relatively large $D_{0}=0.51$ mm drop. If $\delta_{l0}=\delta_{L}^{\text{veq}}=0$ then the drop in NoEqNoDiff would trivially maintain δ=0 (not shown).

Experiments Control and OnlyEq for this relatively depleted environment also are initially depleted by the exchange. After the initial depletion, the drops in the experiments enrich by equilibrium distillation and/or diffusive distillation, respectively, for each experiment. The depleted environmental vapor also shifts the end points by −1‰. As before, OnlyEq approaches the end point for n=0, and Control approaches the end point for n=1.

The depletion by the exchange process, opposite the enrichment by the equilibrium and diffusion processes, results in non‐monotonic adjustment that depends on the drop size (purple, Figure 11). Smaller drops exchange faster, reach a minimum isotope ratio, then enrich faster by isotopic equilibrium and diffusive distillation. Larger drops fall fast and adjust relatively slowly. Thus, the isotope ratio trajectories cross for drops of different initial sizes.

## 5 Conclusion

This study is motivated by the need to understand fractionation and vertical transport of water in trade cumulus boundary layers, to quantify cloud feedbacks and the amount effect. Rain both transports and fractionates water isotopes. Our model of rain evaporation is used to assess isotopes in rain and its reevaporated vapor. Predictions of the model are important to the fate of water and the interpretation of isotope measurements of rain and vapor in the subcloud boundary layer: Rain reevaporation enriches subcloud vapor, explaining the source of enriched vapor cold pools. Small drops in the DSD evaporate completely, explaining the vapor enrichment. The role of molecular diffusion increases as drops shrink. Modeling this process shows that, before they completely evaporate, very small drops enrich more than previously thought.

We used the Craig and Gordon (1965) equation to model the exchange of hydrometeors (raindrops and cloud droplets) with the environment, equilibrium evaporation, and turbulent and molecular diffusion. Simplified experiments illustrate the different effects of exchange, equilibrium fractionation, and molecular diffusive (kinetic) fractionation. Net evaporation distills the isotopes of drops and vapor due to equilibrium and diffusive fractionation. This combination of effects has a profound effect on the isotope fractionation, strongly enriching drops compared to equilibrium mixing or Rayleigh distillation.

Evaporation and exchange with the surrounding vapor enrich the drop *liquid*. When rain evaporates below cloud base, the drops approach an equilibrium temperature and isotope ratio by exchange of enthalpy and vapor with the surrounding air. Results in an isotope ratio equilibrium that differs significantly from the equilibrium of vapor enclosed over a liquid surface. The nearness of the drops to this equilibrium with their surroundings results in surface precipitation whose isotope ratio is mainly determined by the subcloud vapor. This exchange of the rain with relatively enriched subcloud vapor explains the observed correlation of the precipitation isotope ratio to the local surface humidity (Crawford et al., 2017).

On the other hand, the hydrometeor source of *vapor* to the air is nearly the original liquid, because the bulk of the water vapor produced by evaporation comes from individual drops that lose a large fraction of their mass to evaporation. The divergence of the rain flux yields the vapor and isotope source for evaporative downdraft cores. Evaporation of the drops enriches the subcloud vapor deuterium ratio by 12‰ per 1 mm of rain accumulation. The temporary enrichment of vanishing drops, and its enhancement by diffusion, is an interesting flourish that transports enriched water downward but does not ultimately change the isotope ratio of the water that becomes vapor.

A broad range of drop sizes in the DSD further enriches the isotope ratio of the evaporated water. The precocious complete evaporation of small drops enriches $\delta_{D}$ of the resulting vapor by +10‰ compared to a single drop. Because small drops evaporate quickly and completely, cumulative vapor evaporated from realistic drop size distributions become enriched quickly to δ = –20‰ even with a large fraction (f≈0.95) of the rain remains.

Our model extends the work of Stewart (1975) by accounting for the DSD and resolving the kinetic effects associated with diffusion in the drop‐vapor laminar boundary layer. Assuming a spherical laminar layer around the drop, the diameter‐dependent ratio of molecular and turbulent vapor diffusion is parameterized to match previous experimental results. This diffusion model predicts relatively stronger molecular diffusion and kinetic enrichment for small drops. Drops become more enriched as they vanish, by 42% more than predicted by the constant molecular diffusivity ratio, $n=\rho_{m}/\rho \approx 0.58$, previously measured for drops larger than 1 mm diameter. Laboratory and field observations are needed to test our parameterization over a wider range of drop diameters.

Modeling the diffusion has several broader applications: it yields the humidity as a function of distance from an isolated drop, and it can be used to account for the effect of diffusive conduction and evaporation on drop temperature. Small evaporating sea spray droplets (D=0.01–1.0 mm) are also enriched by these kinetic effects, suggesting that coincident measurements of the stable isotopes in atmospheric water vapor, the ocean, and sea spray could constrain the ratio of sea spray evaporation to surface evaporation.

Our simple reevaporation model is physically general and can be used for different warm rain cases, drop size distributions, and environmental humidity profiles. The diameter of evaporating drops has an elliptical dependence on height for locally linear relative humidity profiles. Our model yields the height at which drops completely evaporate. Because all their liquid is converted to vapor, completely evaporated drops have a simple effect on the isotopes. Drops smaller than 0.5 mm evaporate completely before falling 700 m (to the surface) in typical subtropical marine boundary layer conditions. The early and complete evaporation of the smaller drops in the rain size distribution enriches the vapor produced by rain evaporation.

This microphysical raindrop isotope model complements fluid dynamical models of the atmosphere. The isotope concentration can be diagnosed algebraically from the rain drop mass lost to evaporation. It can be evaluated offline or in a single column model (Bony et al., 2008). Because the model can be evaluated algebraically on fluid dynamical model time steps without resorting to shorter microphysical time steps, it can be implemented within isotope‐enabled atmospheric circulation models and LES to understand how using a size‐resolved representation of rain evaporation would affect isotopic composition.

## Data Availability

The NOAA PSL surface meteorology data (NOAA Physical Sciences Laboratory, 2020), the water isotope analyzer data (Bailey & Noone, 2021), and the rainwater isotope analysis data (Quiñones Meléndez et al., 2022) from the *Ronald H*. *Brown* and EUREC4A‐ATOMIC field experiment are accessible from their respective references. Computational notebooks written in the Julia language (Bezanson et al., 2017) are publicly archived (de Szoeke, 2024).

## Appendix A The Laminar Length Scale $l_{\nu }$

In a laminar layer of thickness $l_{\nu }$ the rare water vapor isotopologue diffuses slower than the abundant vapor. Beyond this laminar layer, eddy diffusivity dominates, which diffuses both isotopologues equally. We parameterize the laminar layer thickness as

$$l_{\nu }=\nu /U$$

with kinematic viscosity ν and velocity scale U.

The velocity scale U is the speed of the drop relative to the surrounding air. U is usually found from the fall velocity of the drop. Small droplets (Reynolds number Re=DU/ν<1) fall with the weak velocity Stokes solution. Turbulent air velocities also accelerate the droplets, resulting in relative velocities.

We first neglect gravity and consider a velocity component of the droplet u suspended in air with velocity $u_{\mathrm{air}}$. The relative speed in this component is $u^{\prime }=u_{\mathrm{air}}-u$. The inertia of the drop is balanced by the drag from the surrounding air:

$$m\frac{du}{dt}=\frac{\pi }{6}{\hat{\rho }}_{l}D^{3}\frac{du}{dt}=\frac{\pi }{8}\hat{\rho }D^{2}C_{D}\left(u_{\mathrm{air}}-u\right)|u_{\mathrm{air}}-u|$$

so

$$\frac{du}{dt}=\frac{3}{4}\frac{\hat{\rho }}{{\hat{\rho }}_{l}}\frac{C_{D}}{D}\left(u_{\mathrm{air}}-u\right)|u_{\mathrm{air}}-u|$$

Multiplying the equation of motion by the relative velocity $u^{\prime }$, we write the eddy kinetic energy $\bar{u^{\prime 2}}/2$ equation. Neglecting correlations between anomalies u and $u_{\mathrm{air}}$,

$$\frac{d}{dt}\frac{\bar{u^{\prime 2}}}{2}=-k|u^{\prime 3}|=-k|\bar{u_{\mathrm{air}}^{3}}-\bar{u^{3}}|$$

with

$$k=\frac{3}{4}\frac{\hat{\rho }}{{\hat{\rho }}_{l}}\frac{C_{D}}{D}.$$

Though the turbulence can drive temporary velocity anomalies, mean relative velocity kinetic energy strictly dissipates by drag. Its decay rate goes to zero when the third moments of air and drop velocity balance: $\bar{u_{\mathrm{air}}^{3}}=\bar{u^{3}}$. Though the third moments are not equivalent to second moments, we assume the third moments are equal when the variances are equal $\bar{u_{\mathrm{air}}^{2}}=\bar{u^{2}}$.

The turbulent kinetic energy TKE is proportional to any one component of the air velocity,

$$\bar{u_{\mathrm{air}}^{2}}=2TKE/3.$$

The air and drop velocities are uncorrelated so

$$\bar{u^{\prime 2}}=\bar{{\left(u-u_{\mathrm{air}}\right)}^{2}}=\bar{u^{2}}+\bar{u_{\mathrm{air}}^{2}}=2\bar{u_{\mathrm{air}}^{2}}=4TKE/3.$$

All three components of the velocity are geometrically orthogonal, and the mean fall velocity $U_{\text{fall}}$ is statistically orthogonal, so their magnitudes add in quadrature,

(A1)
$$U={\left(4TKE+U_{\text{fall}}^{2}\right)}^{1/2}.$$

This drop‐relative velocity is used for calculation of the laminar length scale. The contribution of the TKE is small, compared to the fall velocity.

## Appendix B Temperature and Humidity of the Drop and Environment

### B1 Saturation Deficit

Drops nearly approach the wet bulb temperature (Stewart, 1975) in an environment of uniform specific humidity q and adiabatic temperature. We first rewrite Equation 2 by noting that the kinematic vapor diffusivity in air is $K_{va}=k_{va}/\hat{\rho }$ and $\Delta {\hat{\rho }}_{v}/\hat{\rho }=\Delta q=q_{s}\left(T_{r}\right)-q_{a}$ so

$$\frac{dD}{dz}=\frac{4f_{v}k_{va}}{DU_{\text{fall}}{\hat{\rho }}_{l}}\Delta q.$$

We linearize the local saturation specific humidity $q_{s}\left(T_{r}\right)$ of the drop about a representative temperature $\tilde{T}$. The humidity of the air $q_{a}=q_{s}\left(T_{0}\right)$ is saturated at the cloud base at temperature $T_{0}$. The linearization for Δq about $\tilde{T}$ is

$$\Delta q=q_{s}\left(T_{r}\right)-q_{a}={\left(\frac{\partial q_{s}}{\partial T}\right)}_{\tilde{T}}\left(T_{r}-\tilde{T}\right)-q_{a}.$$

The drop temperature is very near the wet bulb temperature of the environment, yielding

$$\Delta q={\left(\frac{\partial q_{s}}{\partial T}\right)}_{\tilde{T}}\Gamma_{w}\left(z^{\prime }-\tau_{w}U_{\text{fall}}\right)$$

where $z^{\prime }=z_{0}-z>0$ is the displacement fallen from cloud base, $\tau_{w}$ is the evaporative adjustment time scale of the drop (B2 in Appendix B), and

$$\Gamma_{w}=\Gamma_{\text{ad}}{\left(1+L{\left(\partial q_{s}/\partial T\right)}_{\tilde{T}}/c_{p}\right)}^{-1}$$

is the linearized lapse rate of wet bulb potential temperature. The adiabatic lapse rate is $\Gamma_{\text{ad}}=g/c_{p}$. This approximates the linear profile of RH used by Sarkar et al. (2023). The heat transport length scale $\lambda =\tau_{w}U_{\text{fall}}$ is the small downward displacement of cooler wet bulb temperature by the falling drop. It is largest (32 m) for the largest drops at cloud base (B2 in Appendix B), which is considerably smaller than turbulent displacements. The turbulence and the small heat transport displacement shall be neglected.

### B2 Drop Temperature

A falling drop evolves toward a wet bulb temperature. Its temperature equation is

(B1)
$$\frac{dT}{dt}=-\frac{12f_{T}K_{Ta}\hat{\rho }c_{p}}{D^{2}{\hat{\rho }}_{l}c_{w}}\left[\left(T-T_{a}\right)+\frac{L}{c_{p}}\frac{f_{v}K_{va}}{f_{h}K_{Ta}}\left(q_{s}\left(T\right)-q_{a}\right)\right],$$

with kinematic conductivity $K_{Ta}=k_{Ta}/\left(\hat{\rho }c_{p}\right)$, vapor diffusivity in air $K_{va}$ ($m^{2}$ $s^{-1}$), density ${\hat{\rho }}_{l}$ and specific heat of liquid water $c_{w}$, drop diameter D, and specific humidity $q={\hat{\rho }}_{v}/\hat{\rho }$. Radiative heating and solution and surface tension effects are neglected. For typical temperature and pressure below the cloud, $K_{va}/K_{Ta}=1.16$.

The conventional wet bulb temperature $T_{w0}$ is defined by

$$c_{p}\left(T_{w0}-T_{a}\right)=-L\left(q_{s}\left(T_{w0}\right)-q_{a}\right).$$

This applies to a well‐ventilated interface, but inspection of Equation B1 shows that the equilibrium wet bulb temperature of a drop is slightly modified by the ratios, of vapor to temperature, of ventilation factors, and diffusivities,

(B2)
$$T_{w}=T_{a}-\left(L/c_{p}\right)\left(f_{v}K_{va}/f_{h}K_{Ta}\right)\left(q_{s}\left(T_{w}\right)-q_{a}\right).$$

It still depends mostly on the environment $T_{a},q_{a}$, and slightly on the diameter through the ventilation factors. The ratio of the ventilation factors $f_{v}/f_{h}$ is nearly unity (Abraham, 1962). This effect is parameterized in the body of the paper by $n=\rho_{m}/\rho$. The diffusivity ratio $K_{va}/K_{Ta}=1.16$ results in a 6% increase of the air‐drop temperature difference $T_{a}-T_{w}$ compared to $T_{a}-T_{w0}$.

The wet bulb temperature is practical to measure, yet it cannot be found analytically from air temperature and humidity because the empirical equilibrium humidity $\left(q_{s}\left(T\right)\right)$ cannot be inverted analytically. Corpart et al. (2023) solve for the drop temperature by approximating the equilibrium humidity by a quadratic. Calculating the derivative $\partial q_{s}/\partial T$) by automatic differentiation, we solve Equation B2 numerically within $10^{-3}$ K in two iterations of Newton's method.

How fast does the drop approach $T_{w}$? The time scale for temperature conduction is

$$\tau_{T}={\left(\frac{12f_{h}K_{Ta}\hat{\rho }c_{p}}{D^{2}{\hat{\rho }}_{l}c_{w}}\right)}^{-1}.$$

The wet bulb adjustment is faster than temperature conduction because of evaporative heating. Linearizing the saturation specific humidity about the wet bulb temperature, $T_{w}$, $q-q_{s}\left(T_{w}\right)={\left(\partial q_{s}/\partial T\right)}_{T_{w}}\left(T-T_{w}\right)$, we find the temperature equation adjusts as follows:

$$\frac{dT}{dt}=-\left(T-T_{w}\right)\left(1+\beta \right)/\tau_{T}=-\left(T-T_{w}\right)/\tau_{w}$$

with

$$\beta =\frac{L}{c_{p}}\frac{f_{v}K_{va}}{f_{T}K_{Ta}}{\left(\frac{\partial q_{s}}{\partial T}\right)}_{T_{w}}\approx \frac{L}{c_{p}}{\left(\frac{\partial q_{s}}{\partial T}\right)}_{T_{w}}.$$

The conductive‐evaporative temperature adjustment time scale for the drop is

(B3)
$$\tau_{w}=\tau_{T}/\left(1+\beta \right),$$

which is (1+β)∼2.8 times shorter than $\tau_{T}$ at T= 290 K. This time scale will be used to compute the displacement and temperature adjustment as drops fall.

### B3 Fall Transport

Drops are cooler than the local wet bulb temperature of their environment, because they fall from the cooler environment aloft. In the drop's frame of reference, $T_{w}$ of the environment warms as the drop descends

$${\left(\frac{dT_{w}}{dt}\right)}_{\text{fall}}=+U_{\text{fall}}\Gamma_{w}$$

with lapse rate $\Gamma_{w}=dT_{w}/dz<0$ and downward velocity $U_{\text{fall}}=-dz/dt>0$. The equilibrium depression from the wet bulb temperature $\Delta T=T-T_{w}$ is solved by balancing this falling source and the evaporative‐conductive temperature source above:

$$0=\frac{d}{dt}\Delta T={\left(\frac{d}{dt}\Delta T\right)}_{\text{evap}-\text{cdct}}+{\left(\frac{d}{dt}\Delta T\right)}_{\text{fall}}=-\Delta T/\tau_{w}+U_{\text{fall}}\Gamma_{w}.$$

The drop temperature T is cooler than the surrounding wet bulb temperature $T_{w}$ because the drop T adjusts to the environmental $T_{w}$ over an integral length scale $\lambda =\tau_{w}U_{\text{fall}}$, and

$$T=T_{w}+\lambda \Gamma_{w}.$$

The adjustment length scale λ is largest for large drops, reaching ∼30 m for drops D>1 mm. This is smaller than displacements by turbulent eddies. This diagnoses a 1.0 mm drop has temperature $T=T_{w}-0.2$ K and 0.1 mm drop has $T=T_{w}-0.001$ K. The difference $T-T_{w}$ is also small in numerical integrations of Equation B1. The temperature adjustment due to this length scale has been neglected in the body of the paper.
